# Multi-Objective Optimization of Injection Molding Process Parameters of Car Lamp Shell Based on Grey Correlation Analysis

**DOI:** 10.3390/polym17111524

**Published:** 2025-05-29

**Authors:** Ruixia Shan, Anqin Liu, Sen Jia, Changyou Liu, Wenguang Yang

**Affiliations:** 1 School of Mechanical and Electrical Engineering, Yantai Institute of Technology, Yantai 264005, China; liuan_821@163.com (A.L.); jiasen0504@163.com (S.J.); changyou660@126.com (C.L.); 2 School of Electromechanical and Automotive Engineering, Yantai University, Yantai 264005, China

**Keywords:** injection molding process parameter, grey relational analysis, CRITIC method, multi-objective optimization

## Abstract

In order to improve the injection molding quality of the car lamp shell, orthogonal test, signal-to-noise ratio, gray correlation analysis, and CRITIC weight method were used to analyze the influence of mold temperature, melt temperature, injection time, velocity to pressure control, pressure holding pressure and pressure holding time on the shrinkage index and the total deformation of warpage, and fully consider the difference and correlation between the evaluation parameters. The multi-objective optimization is transformed into single-objective optimization, and the optimal parameter set is obtained. The experimental results show that, compared with the initial analysis results, the indentation index of the headlight shell is reduced by 33.95%, the total warpage deformation is reduced by 13.99%, and the forming quality of the headlight shell is improved. The research results provide a theoretical reference value for multi-objective optimization of plastic injection molding process parameters.

## 1. Introduction

With the improvement of automobile aesthetics, the surface quality and dimensional accuracy of injection molding parts such as car lights, bumpers, and interiors are increasingly required, and research on the molding quality of injection molding parts is also widely concerned. In the process of injection molding, the injection molded parts will be affected by internal and external factors in the mold cavity, which easily produce quality defects such as warping deformation, weld marks, shrinkage marks, uneven shrinkage, flash, and so on. The quality of injection-molded parts depends not only on the material properties and mold structure but also on the molding process parameters. In order to obtain high appearance quality and high precision dimensional control, it is necessary to optimize the process parameters that affect the quality of injection molding. Pang et al. used Moldflow to simulate the injection molding process of automotive headlights, obtained the injection molding process parameters through orthogonal test analysis, and optimized the warping deformation [1]. Yin et al. used Moldflow for mold flow analysis of the vehicle lamp body, adopted the optimal Latin hypercube test design method to select analysis samples, built an approximate response surface model based on the analysis results, and used the ASA algorithm to optimize process parameters, reducing the warpage deformation [2]. Ding et al. used Moldflow software to simulate and analyze the molding process of the rear cover of headlights, optimize the cooling circuit, and effectively reduce the warping deformation caused by uneven shrinkage through orthogonal test analysis and optimization [3].

Due to the existence of multiple injection defects in the injection molding process of the car lamp shell, multiple quality objectives are required as evaluation parameters for multi-objective optimization in the optimization study of injection molding process parameters [4]. Li Chunxiao et al. took volume shrinkage rate, X-direction warping deformation, and Z-direction warping deformation as evaluation indexes for electrostatic detection boxes, constructed a generalized neural network (GRNN) model between injection molding process parameters and evaluation indexes through orthogonal tests, and combined with fast non-dominated sorting algorithm (NSGA-II) to obtain the best injection molding process parameters and reduce injection molding defects [5]. Ouyang Yu et al. designed a 5-factor and 4-level orthogonal test on the injection molding process of the funboard. With the maximum warpage deformation, volume shrinkage rate, and shrink mark length as evaluation parameters, the signal-to-noise ratio and normalization of the test results were processed, the gray correlation coefficient was calculated, and the weight of each evaluation parameter was obtained by principal component analysis (PCA). The multi-objective optimization was transformed into a single-objective gray correlation degree (GRA) analysis, which obtained the best injection molding process parameter combination of plastic parts and improved the injection molding quality [6]. Wang Xiaodong et al. took the two-color plastic bowl as an example, carried out signal-to-noise ratio, dimensionless processing, and gray correlation coefficient calculation on the orthogonal test results, calculated the total warpage deformation and the weight of the average volume shrinkage of the second shot by entropy weight method, transformed the multi-objective optimization into single-objective optimization, and achieved remarkable optimization effect [7].

The surface quality and dimensional accuracy of the car lamp shell are greatly affected by the shrinkage mark and warpage deformation during the injection molding process. Therefore, in order to improve the molding quality of the car lamp shell, Moldflow software was used to analyze the injection process, combined with the existing research methods and theories. The operating parameters such as mold temperature, melt temperature, injection time, velocity to pressure control, pressure holding pressure, and pressure holding time were used as experimental factors, and a 6-factor 5-level orthogonal test was established. The shrinkage index and the total warpage deformation were used as evaluation parameters. The differences and correlations between the evaluation parameters were fully considered. Criteria Importance Through Intercriteria Correlation (CRITIC) was used to calculate the weight of evaluation parameters, and the grey correlation analysis was used to find the injection molding process scheme to improve the quality of plastic parts.

## 2. Product Model and Initial Injection Molding Simulation Analysis

### 2.1. Car Lamp Shell Model and Grid Division

**Figure 1:**
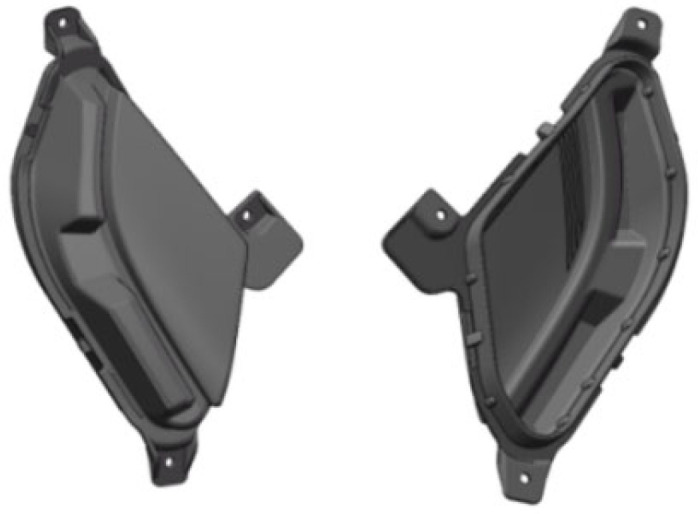
Model of car light.

The headlight shell model of this study is shown in Figure 1. The dimensions of the headlight shell are 172 mm × 130 mm × 105 mm, the average wall thickness of the headlight shell is about 2.5 mm, and the wall thickness at the surrounding convex is about 4–5 mm.

CADdoctor EX 6.0 was used to check the model, repair defects such as missing surfaces and gaps, and simplify features such as rounded corners and chamfers that do not affect the main structure so as to improve the quality of grid division. The model converted from CADdoctor is imported into Moldflow, and the Dual Domain mesh type is used to grid the lamp shell model. The mesh defects are repaired according to the grid information to obtain a better grid model. The mesh side length is 1.30 mm, the maximum aspect ratio is 17.60 mm, the minimum aspect ratio is 1.16 mm, and the average aspect ratio is 1.77 mm. Free edge, multiple edges, intersecting element, incorrectly aligned element, and completely overlapping element are all 0, and the matching percentage is 89.2%, which meets the requirements of mode flow analysis.

### 2.2. Setting of Forming Materials

The shell material of the car lamp is PP + TD30, which is the base material of the lamp shell, and TD30 is 30% talc added to the material, which can improve the rigidity of the plastic parts. The molding material chosen is PP material produced by Lotte Chemical, which is located in Seoul, South Korea. The brand is A-373, and the recommended process parameters are shown in Table 1.

### 2.3. Initial Injection Molding Simulation Analysis

**Figure 2:**
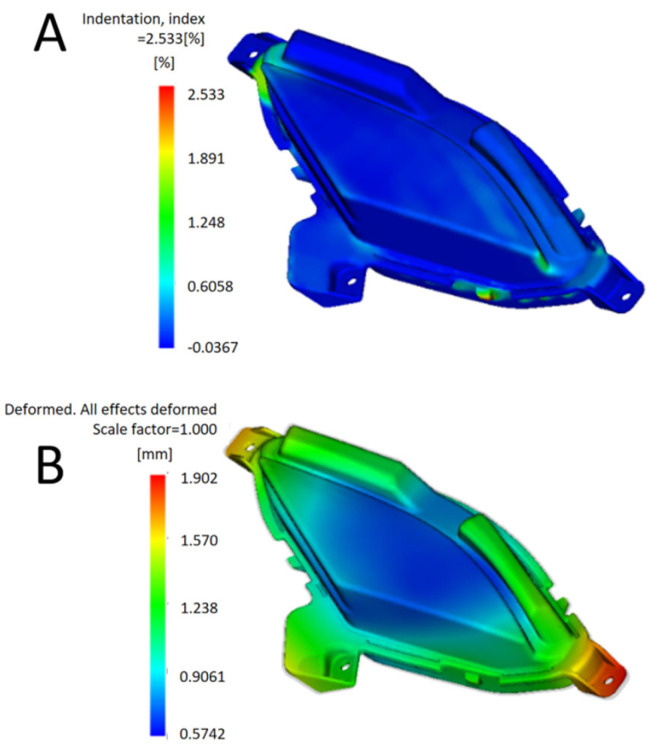
(**A**) Indentation index. (**B**) Warping total deformation.

The “molding window” and “filling + holding pressure” analyses were carried out to determine the molding process factors and ranges that affect the production of qualified products. Mold temperature was set to 50 °C, melt temperature to 230 °C, injection time to 0.8 s, velocity to pressure control to 98%, pressure holding pressure to 15 MPa, and pressure holding time to 5 s. The analysis sequence of “cooling + filling + pressure holding + warping” was selected for injection simulation analysis. The analysis results are shown in Figure 2, and the shrinkage index was 2.533%. The total deformation of warping is 1.902 mm, and the quality of injection molding is poor.

## 3. Test Scheme

### 3.1. Orthogonal Experimental Design

In the injection molding of plastic parts, the selection and control of injection molding process conditions are the key factors to ensure the molding efficiency and the quality of plastic parts. Orthogonal tests can select representative tests from comprehensive tests according to orthogonality, find the main factors affecting quality, and obtain the best parameters for quality control through the analysis of representative test results [10].

Molding process conditions: mold temperature (A), melt temperature (B), injection time (C), velocity to pressure control (D), pressure holding pressure (E), and pressure holding time (F) were selected as test factors. Orthogonal tests with six factors and five levels were designed using orthogonal table L25 (5^6^), and Table 2 showed orthogonal test factors and levels.

### 3.2. Orthogonal Test Results

If there are defects such as pores, shrinkage marks, warping, and shrinkage, the appearance quality and dimensional accuracy of plastic parts will be affected. The fewer shrinkage marks and the smaller the warping deformation, the higher the quality of plastic parts, so the shrinkage index (X/%) and the total deformation of warping (Y/mm) are selected as the evaluation parameters. Moldflow software was used to simulate the injection molding process of 25 groups of orthogonal tests, and the test results were recorded in Table 3.

## 4. Multi-Objective Grey Correlation Analysis

### 4.1. Signal-to-Noise Ratio Processing

In the simulation analysis of the injection molding process, the analysis results are affected by many factors such as grid density and type, analysis solution method, and iteration method, and the experimental results are uncertain. In the statistical analysis of data, the SNR can retain effective information and filter the ineffective interference information, making the analysis result more accurate. According to the different measurement indicators, the SNR has visual, small, and large characteristics [11]. In order to eliminate the influence of random interference factors in the orthogonal test, the signal-to-noise ratio of evaluation parameter test results of plastic surface quality was processed, and the denoising results of each index test value of plastic surface quality were used for data analysis. The shrinkage index is calculated when the local pressure of each unit drops to 0 during the pressure holding period, reflecting how much material is still the solution and not the pressure holding; the higher the shrinkage index value, the higher the potential shrinkage, and the greater the possibility of shrinkage marks on the surface of the workpiece. For this reason, the smaller the shrinkage index value, the better; that is, the shrinkage index has the characteristics of hope. Warping deformation is the distortion of the shape of the plastic parts, and the warping uneven, deviating from the shape accuracy requirements of the plastic parts, is one of the common defects in the production of injection molding, affecting the appearance and performance requirements of the plastic parts, so the smaller the total deformation of the warping, the better. The signal-to-noise ratio (SNR) was processed using Formula (1) with low expectation characteristics, and the signal-to-noise ratio processing results of indentation index and total warp deformation were recorded in Table 4.

(1) $$\alpha =-10\mathrm{lg}(\frac{1}{n}\sum_{a=1}^{n}{X_{a}}^{2})$$

In the formula, α represents the value of the signal-to-noise ratio after processing; *X_a_* represents the value of the repeat test; *n* represents the number of repeated tests, *n* = 1.

### 4.2. Calculation of Grey Relational Degree

Grey correlation analysis is a systematic scientific theory that can judge the closeness of the relation by comparing the geometric shape similarity between the sequence and the reference sequence and transforming the multi-objective optimization into single-objective optimization. According to the grey correlation degree of injection molding process surface quality evaluation parameters, the multi-objective optimization problem is transformed into a single objective comparison grey correlation degree problem so as to judge the strength of the relationship between process parameters and evaluation parameters and to achieve multi-objective optimization and determine the optimal injection molding process parameter combination [12].

#### 4.2.1. Non-Dimensional Processing

The values of indentation index and total warp deformation after signal-to-noise ratio processing are used as the original data for test data analysis, and the data normalization method is applied to the original data for data standardization so as to avoid the phenomenon of inaccurate analysis results due to the different dimensions of indentation index and total warp deformation, eliminate the dimensions between each evaluation parameter, and obtain dimensionless data. Dimensionless processing allows all data to be compressed within the range of [0, 1], keeping the mathematical units consistent between the data, and the larger the indicator number, the better. The dimensionless values obtained by using Formula (2) are shown in Table 4.

(2) $$\beta_{i}=\frac{\alpha_{i}-\alpha_{min}}{\alpha_{max}-\alpha_{min}}$$

where $\beta_{i}$ represents the dimensionless value of the signal-to-noise ratio; $\alpha_{i}$ represents the signal-to-noise ratio after the numerical conversion of the i test; $\alpha_{max}$ represents the maximum signal-to-noise ratio of each test parameter in Table 4; $\alpha_{min}$ represents the minimum signal-to-noise ratio for each test parameter in Table 4.

#### 4.2.2. Grey Correlation Coefficient

The grey correlation coefficient is an intermediate transition value, which represents the relationship between the comparison series (dimensionless signal-to-noise ratio data) and the reference series of evaluation parameters such as indentation index and total warp deformation. According to the grey system theory, the reference series is selected as β_0_ = {1,1}, the dimensionless signal-to-noise ratio of each evaluation parameter in Table 4 is substituted into Formula (3), and the grey correlation coefficient corresponding to the shrinkage index and the total deformation of warpage is calculated, as shown in Table 4.

(3) $$\gamma_{ij}=\frac{\underset{i}{\min}\underset{j}{\min}\left|\beta_{0}\left(j\right)-\beta_{i}\left(j\right)\right|+\rho \underset{i}{\max}\underset{j}{\max}\left|\beta_{0}\left(j\right)-\beta_{i}\left(j\right)\right|}{\left|\beta_{0}\left(j\right)-\beta_{i}\left(j\right)\right|+\rho \underset{i}{\max}\underset{j}{\max}\left|\beta_{0}\left(j\right)-\beta_{i}\left(j\right)\right|}$$

where γ_ij_ represents the gray correlation coefficient of the JTH evaluation parameter of the i experiment, i = 1, 2, …, 25, j = 1, 2; β_0_(j) represents the reference value of the JTH evaluation parameter; β_i_(j) represents the dimensionless value of the signal-to-noise ratio of the JTH evaluation parameter of the i experiment; ρ represents the resolution coefficient, whose range is 0~1, generally take ρ = 0.5.

#### 4.2.3. Grey Correlation Degree

The grey correlation degree is the weighted average of the grey correlation coefficient. The weight values of the shrinkage index and the total warpage deformation are calculated using the CRITIC method. The grey correlation degree of each test order of the surface quality of plastic parts can be further calculated by using Formula (4), and the results are shown in Table 4.

(4) $$Ƞ=\sum_{j=1}^{n}W_{j}\gamma_{ij}$$

where Ƞ represents the gray correlation degree; $W_{j}$ represents the weight of the JTH evaluation parameter; *n* Indicates the number of evaluation parameters.

### 4.3. CRITIC Method to Calculate Weight

In the grey correlation analysis of multi-objective optimization transformed into single-objective optimization, the weight of each evaluation parameter represents the importance and influence of the evaluation parameters, and the objectivity and accuracy of the weight directly affect the final optimization result. Although the subjective weighting method gives weight through scientific analysis, it is greatly influenced by the evaluator, and the accuracy and credibility of the weighting result are not high. The principal component analysis is to condense multiple indicators into a few general indicators and calculate the weight by extracting the information from the general indicators, but some information will be lost in the process of dimensionality reduction. The entropy weight method can use the information carried by the data to calculate the weight, which is a relatively objective method to obtain the weight of the evaluation parameters, but it mainly considers the fluctuation of the data and ignores the correlation between the evaluation parameters. In the process of injection molding, the shrinkage marks are affected by factors such as poor cooling shrinkage, geometric characteristics of the parts, injection position, cavity filling conditions, and the warping deformation is affected by factors such as uneven cooling, uneven shrinkage, shape of the parts, and mold structure. There are often differences, as well as correlations between evaluation parameters such as shrinkage index and total warp deformation. Therefore, the CRITIC method is proposed to calculate the weight to mine the information contained in evaluation parameter data more objectively and comprehensively and improve the rationality and objectivity of weighting.

The weight method of CRITIC makes use of the objective attributes of the data itself and assigns weight objectively according to the volatility and correlation of the data. Its idea lies in two indicators, namely, the volatility indicator and the conflict indicator. In weight calculation, it is necessary to carry out data standardization and multiply volatility indicators and conflict indicators to obtain the final weight [13]. The steps to calculate the weight using the CRITIC method are as follows:

1. Obtain data

There are m objects to be evaluated and n evaluation parameters to form the original data matrix X.

(5) $$X=\left(\begin{array}{ccc} x_{11} & \ldots & x_{1n}\\ \vdots & \ddots & \vdots \\ x_{m1} & \ldots & x_{mn} \end{array}\right)$$

2. Data standardization

Avoid the interference caused by dimensional problems so that the data can be measured by a uniform standard. The smaller the shrinkage index and the total deformation of warp, the better, and the reverse processing is performed using Formula (6).

(6) $$x_{ij}=\frac{{max\left\{x_{ij}\right\}-x}_{ij}}{max\left\{x_{ij}\right\}-min\left\{x_{ij}\right\}}$$

where *i* indicates the serial number of the evaluation object, *i* = 1, 2, …, m; *j* indicates the number of evaluation parameters, *j* = 1, 2, …, n.

3. Calculate information-bearing capacity

The volatility index is expressed by standard deviation. The larger the standard deviation is, the greater the degree of value change in the evaluation parameter; the more information can be projected, the stronger the evaluation intensity of the index itself will be, and the higher the weight will be. Formula (7) is used for calculation.

(7) $$S_{j}=\sqrt{\frac{\sum_{i=1}^{m}{\left(x_{ij}-\bar{x_{j}}\right)}^{2}}{m-1}}$$

where *S_j_* represents the volatility index of the JTH evaluation parameter; $\bar{x_{j}}$ represents the mean value of the data for the JTH evaluation parameter.

The conflict index represents the correlation between evaluation parameters through the correlation coefficient. If the correlation between evaluation parameters is larger, the conflict between the evaluation parameter and other evaluation parameters will be smaller. The more the same information is reflected, the more repeated the evaluation content will be reflected, and the evaluation intensity of the evaluation parameter will be weakened to a certain extent. The weight assigned to this evaluation parameter should be reduced. Use Formula (8) for calculation.

(8) $$A_{j}=\sum_{i=1}^{m}\left(1-r_{jk}\right)$$

where *A_j_* represents the conflicting index of the JTH evaluation parameter, and *r_jk_* represents the correlation coefficient between the evaluation index *j* and *k*.

4. Information volume

Based on the calculation results of the volatility and conflict of evaluation parameters, the information content of each evaluation parameter is determined by *C_j_*, and the specific calculation formula is shown in Formula (9). The greater the role of evaluation parameters in the whole evaluation index system, the more information *C_j_* will be obtained, and the more weight can be assigned.

*C_j_ = S_j_ × A_j_* (9)

5. Objective weight

The objective weight of the [mathematical formula] evaluation parameter is calculated using Formula (10). The calculation results of the CRITIC weight method are shown in Table 5.

(10) $$W_{j}=\frac{C_{j}}{{\sum_{j=1}^{n}C}_{j}}\;$$

## 5. Optimization of Process Parameters and Verification of Test Results

### 5.1. Optimization of Process Parameters

According to the grey correlation analysis, the greater the grey correlation degree, the better the horizontal characteristics of the process parameters, and the closer the corresponding process parameters are to the optimal. By calculating the average grey correlation degree under different injection process parameters (Table 6), the optimal combination of process parameters can be obtained as A_3_B_1_C_4_D_2_E_5_F_5_, that is, mold temperature is 50 °C, melt temperature is 210 °C, injection time is 1 s, velocity to pressure control is 97%, pressure holding pressure is 21 MPa and pressure holding time is 6 s. The range of grey correlation degrees reflects the degree of influence of each process parameter on the quality objective (the shrinkage index and the total deformation of the warp are minimum). The greater the range, the greater the influence of this parameter on the quality objective. According to the gray correlation degree range of each process parameter in Table 6, the degree of influence on the quality objective is ranked from large to small as holding pressure, melt temperature, holding pressure time, mold temperature, velocity to pressure control, and injection time.

### 5.2. Verification of Test Results

**Figure 3:**
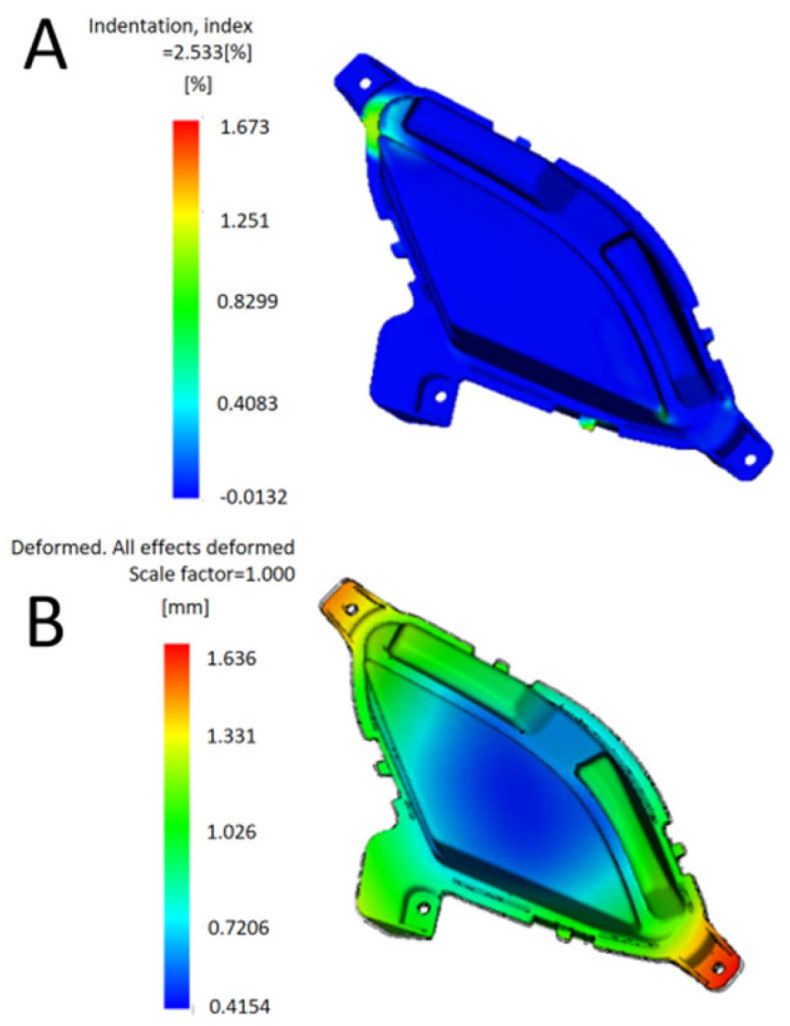
Analysis results of optimization. (**A**) Optimized shrinkage index. (**B**) Total warp deformation after optimization.

The optimal process parameter combination (A_3_B_1_C_4_D_2_E_5_F_5_) obtained by grey correlation analysis was simulated in Moldflow. The results are shown in Figure 3. The optimized shrinkage index is 1.673%, and the total warpage deformation is 1.636 mm, which is 33.95% and 13.99% lower than the initial value, respectively. Compared with the orthogonal test results, they all reached a smaller value. The maximum shrinkage index is small, which does not affect the appearance of the product; the maximum warpage deformation is 0.95% of the overall size, which does not affect the product assembly. The forming quality of the optimized lamp shell is improved, and the overall optimization effect of grey correlation analysis is better. The optimal combination of process parameters provides a reference for the trial production of products [14].

### 5.3. Production Verification

**Figure 4:**
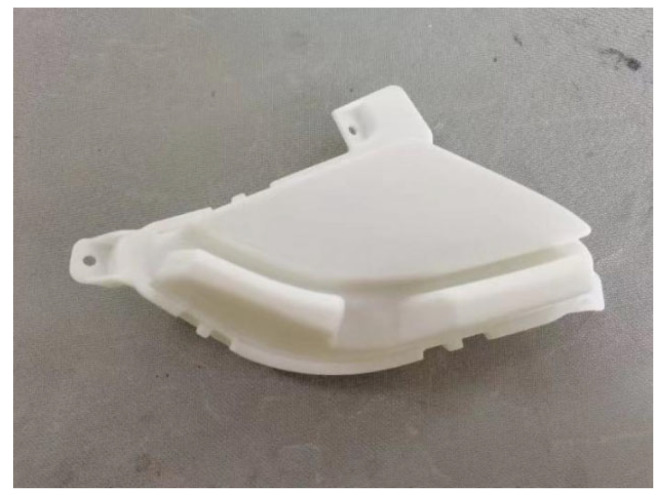
Car light shell products.

**Figure 5:**
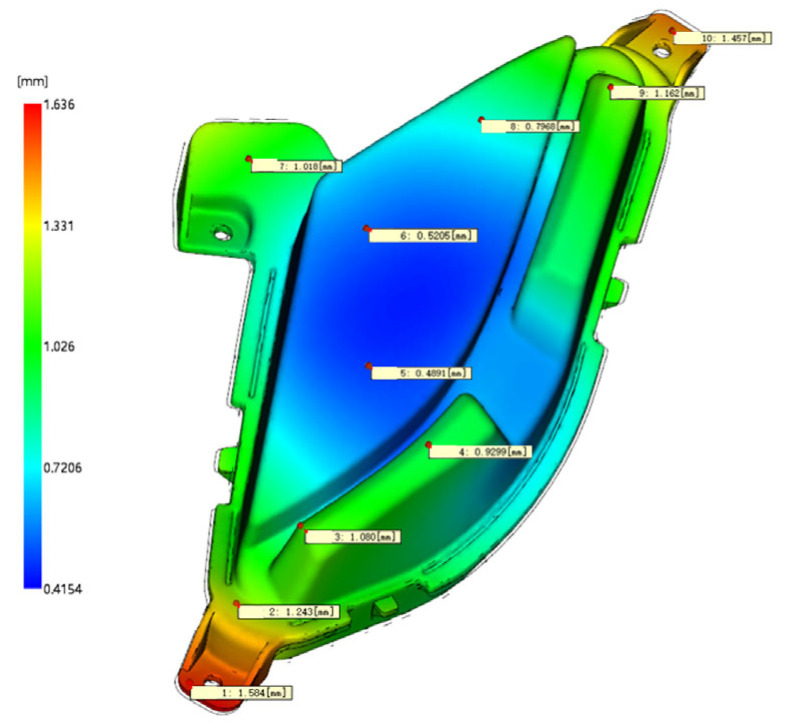
The total warping deformation of the measuring point.

**Figure 6:**
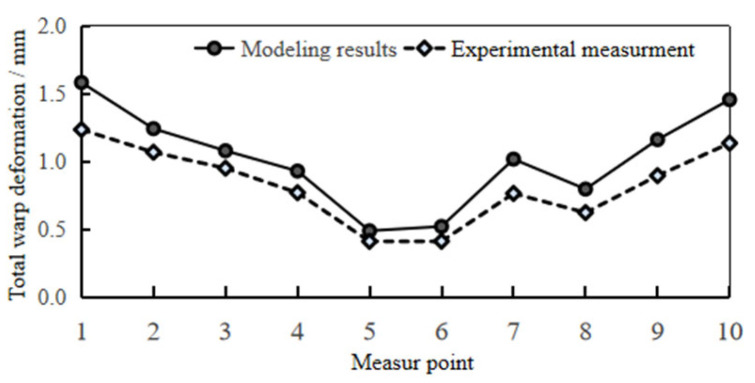
Data of actual measurement and simulation results.

In the trial production of the lamp shell, the optimal process parameters obtained by grey correlation analysis were used for production, and the trial products are shown in Figure 4. The three-dimensional scanner is used to scan and model the test product and compare it with the original three-dimensional model. Ten positions are selected to measure the deformation of the product; the total warping deformation corresponding to 10 positions is extracted from the simulation results, as shown in Figure 5. The data of the actual measured deformation and the simulation results are drawn in Figure 6. The maximum deformation of the test-mold product is 1.236 mm, and the actual measured data are basically consistent with the data of the simulation results. Observe that the surface of the test product is smooth, and there are no obvious shrinkage marks or other defects. It meets the technical requirements and verifies the feasibility and reliability of grey correlation analysis for multi-objective optimization.

## 6. Conclusions

In this study, the lamp housing of vehicle lights was taken as the research object, and an orthogonal experimental design was carried out on the corresponding process parameters affecting the sink mark index and total warpage deformation of plastic parts. Through grey relational analysis, the multi-objective optimization problem was transformed into a single—objective optimization problem regarding the grey relational degree, and the optimal combination of process parameters was determined.

The CRITIC weight method was adopted to determine the weights of the sink mark index and total warpage deformation in injection molding, which were 53.60% and 46.40%, respectively. This method fully considered the differences and correlations among evaluation parameters, providing a comprehensive and objective weighting.

The optimized process parameter combination A_3_B_1_C_4_D_2_E_5_F_5_ was adopted, that is, mold temperature is 50 °C, melt temperature is 210 °C, injection time is 1 s, velocity to pressure control is 97%, pressure holding pressure is 21 MPa, and pressure holding time is 6 s. The simulation analysis and production trial were carried out. The results showed that the shrinkage index was reduced by 33.95%, the total warpage deformation was reduced by 13.99%, and the quality of the injection molded parts was improved. The feasibility of using the combination of the CRITIC weight method and grey correlation analysis to transform the influence of process parameters on the evaluation parameters into the grey correlation degree of the evaluation parameters is verified, which provides a reference for the optimization of the injection molding process.

## Figures and Tables

**Table 1:** Recommended process parameters of materials [8,9].

Processing Property	Parameter
Mold surface temperature/°C	50
Melt temperature/°C	230
MFI/g·(10 min)^−1^	1.62
Melting range/°C	165–180
Mold temperature range/°C	20–80
Melt temperature range/°C	200–280
Ejection temperature/°C	93
Maximum shear stress/MPa	0.26
Maximum shear rate/s^−1^	24,000

**Table 2:** Factors and levels of orthogonal test.

Level	Technological Parameter					
	Mold Temperature (A)/°C	Melt Temperature (B)/°C	Injection Time (C)/s	Velocity to Pressure Control (D)/%	Dwell Pressure (E)/MPa	Dwell Time (F)/s
1	40	210	0.7	96	9	4
2	45	220	0.8	97	12	4.5
3	50	230	0.9	98	15	5
4	55	240	1	99	18	5.5
5	60	250	1.1	100	21	6

**Table 3:** Results of orthogonal test.

Number	Technological Parameter						Evaluation Parameter	
	A	B	C	D	E	F	X	Y
1	1	1	1	1	1	1	3.236	2.429
2	1	2	3	4	5	2	1.875	1.584
3	1	3	5	2	4	3	2.212	1.678
4	1	4	2	5	3	4	2.674	1.791
5	1	5	4	3	2	5	3.040	1.86
6	2	1	5	4	3	5	1.923	1.879
7	2	2	2	2	2	1	2.462	2.019
8	2	3	4	5	1	2	2.931	2.072
9	2	4	1	3	5	3	2.446	1.655
10	2	5	3	1	4	4	2.726	1.708
11	3	1	4	2	5	4	1.691	1.645
12	3	2	1	5	4	5	2.066	1.726
13	3	3	3	3	3	1	2.553	1.918
14	3	4	5	1	2	2	2.881	1.949
15	3	5	2	4	1	3	3.445	2.015
16	4	1	3	5	2	3	2.126	2.029
17	4	2	5	3	1	4	3.371	2.362
18	4	3	2	1	5	5	2.194	1.669
19	4	4	4	4	4	1	2.637	1.819
20	4	5	1	2	3	2	3.057	1.873
21	5	1	2	3	4	2	1.868	1.802
22	5	2	4	1	3	3	2.209	1.894
23	5	3	1	4	2	4	2.649	1.964
24	5	4	3	2	1	5	3.136	2.034
25	5	5	5	5	5	1	2.702	1.738

**Table 4:** Results of grey relational analysis.

Number	Signal-to-Noise Ratio Processing		Dimensionless Processing		Grey Correlation Coefficient		Grey Relational Degree ƞ
	α_x_	α_y_	β_x_	β_y_	γ_x_	γ_y_	
1	−10.200	−7.708	0.088	0	0.362	0.509	0.430
2	−5.460	−3.995	0.855	1	0.827	1	0.907
3	−6.895	−4.495	0.623	0.865	0.627	0.903	0.755
4	−8.543	−5.061	0.356	0.713	0.472	0.809	0.628
5	−9.657	−5.390	0.176	0.624	0.394	0.761	0.564
6	−5.679	−5.478	0.819	0.601	0.791	0.748	0.771
7	−7.825	−6.102	0.472	0.432	0.532	0.668	0.595
8	−9.340	−6.327	0.227	0.372	0.414	0.642	0.520
9	−7.769	−4.375	0.481	0.897	0.537	0.925	0.717
10	−8.710	−4.649	0.329	0.824	0.459	0.876	0.652
11	−4.562	−4.323	1	0.912	1	0.935	0.970
12	−6.302	−4.740	0.719	0.799	0.700	0.861	0.775
13	−8.141	−5.656	0.421	0.552	0.504	0.724	0.606
14	−9.190	−5.796	0.251	0.515	0.424	0.706	0.555
15	−10.743	−6.085	0	0.437	0.333	0.670	0.489
16	−6.551	−6.145	0.678	0.421	0.668	0.663	0.666
17	−10.555	−7.465	0.031	0.065	0.343	0.530	0.430
18	−6.824	−4.449	0.634	0.878	0.636	0.912	0.764
19	−8.422	−5.196	0.376	0.676	0.481	0.789	0.624
20	−9.705	−5.450	0.168	0.608	0.391	0.752	0.559
21	−5.427	−5.115	0.860	0.698	0.832	0.801	0.818
22	−6.883	−5.547	0.624	0.582	0.629	0.739	0.680
23	−8.461	−5.862	0.369	0.497	0.478	0.698	0.580
24	−9.927	−6.167	0.132	0.415	0.378	0.661	0.509
25	−8.633	−4.800	0.341	0.783	0.465	0.851	0.644

**Table 5:** Computation of CRITIC weight method.

Evaluation Parameter	Volatility Indicator S_j_	Conflict Index A_j_	Information Content C_j_	Weight W_j_
X	0.284	0.348	0.099	53.60%
Y	0.246	0.348	0.086	46.40%

**Table 6:** The grey relational proportion of each process.

Level	Grey Relational Proportion					
	Mold Temperature (A)	Melt Temperature (B)	Injection Time (C)	Velocity to Pressure Control (D)	Dwell Pressure (E)	Dwell Time (F)
1	0.657	0.73	0.612	0.616	0.476	0.58
2	0.651	0.677	0.659	0.677	0.592	0.672
3	0.679	0.645	0.668	0.627	0.649	0.661
4	0.608	0.607	0.672	0.674	0.725	0.652
5	0.646	0.582	0.631	0.647	0.800	0.677
Range	0.071	0.148	0.06	0.061	0.324	0.097
Sort	4	2	6	5	1	3

## Data Availability

The original contributions presented in this study are included in the article. Further inquiries can be directed to the corresponding author.
